# How Can Institutional Review Boards Best Interpret Preclinical Data?

**DOI:** 10.1371/journal.pmed.1001011

**Published:** 2011-03-08

**Authors:** James V. Lavery

**Affiliations:** Centre for Research on Inner City Health & Centre for Global Health Research, Keenan Research Centre in the Li Ka Shing Knowledge Institute of St. Michael's Hospital, Toronto, Canada, and Dalla Lana School of Public Health and Joint Centre for Bioethics, University of Toronto, Toronto, Canada

Among the many challenges facing institutional review boards (IRBs) is to predict whether the activities and interventions proposed in a clinical trial protocol are likely to yield net harm or net benefit for trial participants. IRBs take these questions very seriously, and never more so than in the review of first-in-human (FIH) trials, where interpreting findings about risks to humans from animal data requires a leap of faith, regardless of the quality of the available data.

In their paper published this week in *PLoS Medicine* [1], “Predicting harms and benefits in translational trials: Ethics, evidence, and uncertainty,” Jonathan Kimmelman and Alex London argue that decision-makers (which, from the context of their paper, I assume to mean IRB members) pay insufficient attention to threats to validity in preclinical studies and consult too narrow a set of evidence, thereby unnecessarily limiting predictions about risks and potential benefits for humans that they might otherwise be able to make. They advocate greater attention to the quality of preclinical evidence and to research on related agents. These strategies are meant to reduce what they call the “misestimation” of risks or anticipated benefits, which they argue “threatens the integrity of the scientific enterprise, because it frustrates prudent allocation of research resources”[1].

Kimmelman and London's proposal is likely to stimulate a great deal of constructive debate among clinical trialists, regulators, and other members of the research ethics community. In my brief comments here, I will attempt to open this debate by identifying a key aspect of their proposal that is likely to generate particular interest and perhaps even some controversy—that is, their framing of the problem in terms of how effectively decision-makers utilize evidence from preclinical or animal studies. Although IRB members often do not have deep grounding in the subtleties of research design and inferential statistics, it would be wrong to suggest that “misestimation” of risk and potential benefit arises solely from errors by IRB members (or other decision-makers).

## Misestimates of Risk

There is no doubt that IRB members may “misestimate” risk and benefit, in the sense that they may draw erroneous conclusions about the transferability of findings from animal studies to human studies. But it is equally likely that it may often be impossible (or infeasible) to determine when (if ever) the inferences arising from animal studies are truly valid, in the multiple senses suggested. Internal and construct validity, for example, both rely to some degree on the accuracy of the underlying theory, i.e., whether it properly accounts for the relevant mechanisms of action. To understand the dilemma, one need only consider the large number of drugs that are approved for use by regulatory authorities on the basis of some demonstration of efficacy and safety, but whose mechanisms of actions are still unknown or have been poorly understood for many years after approval (e.g., acetominophen, GABA). This issue becomes particularly important in light of Kimmelman and London's proposal to systematize the assessment of preclinical studies from reference classes of compounds as part of the routine due diligence of assessing risk and potential benefit in first in human trials.

The most pressing problem is one of completeness, a specific dimension of construct validity: do the theory and data account for all the relevant elements or mechanisms that might contribute to benefit or harm in humans? This is the “black swan” or “unknown unknowns” problem in FIH trials: What molecular landmines lie beyond our current data or imagination?

## The Black Swan

The widely reported TGN1412 trials in the UK [2] may provide an instructive test case for Kimmelman and London's proposal. Six healthy volunteers were given the test agent, TGN1412 (an immune modulator), which triggered a cytokine storm and subsequent multiple organ failure, even at a fraction of the dose found to be safe in macaque monkeys [2]. Kimmelman and London's proposal could have been useful, in principle, in the TGN1412 trial in that it would have required reviewers to question whether the animal models truly are sufficiently similar to the relevant human systems to permit the right kind of conclusions about safety and potential benefits in humans. One theory about the TGN1412 trials [3] is that the catastrophic effects were mediated by memory B cells, which may have been absent or under-developed in the laboratory animals. The animal data, therefore, would not have been complete in that critical sense. Whether or not this specific theory is correct, it serves well to illustrate that this is not an insight that arises from the animal data themselves. It is a deeper question—precisely the kind that Kimmelman and London are encouraging IRB members to ask, but one for which there may be no obvious trigger. The central shortcoming in construct validity is likely to remain a “black swan” until scrutiny or experience reveals it.

## A Valuable Proposal

Kimmelman and London's proposal is valuable precisely because it encourages IRB members, and other reviewers, to engage with less-familiar challenges and guard against complacency in reviewing risk and benefit data from preclinical studies. But its true potential value likely lies in the extent to which it can forge agreement throughout the research enterprise on the need for more creative approaches to presenting and contextualizing preclinical evidence, and on broadening the base of responsibility for these difficult judgments.

## Abbreviations

FIH: first-in-human
IRB: institutional review board
